# Diversity and Inclusion: Impacts on Psychological Wellbeing Among Lesbian, Gay, Bisexual, Transgender, and Queer Communities

**DOI:** 10.3389/fpsyg.2022.726343

**Published:** 2022-04-29

**Authors:** Alex Siu Wing Chan, Dan Wu, Iris Po Yee Lo, Jacqueline Mei Chi Ho, Elsie Yan

**Affiliations:** ^1^Department of Applied Social Sciences, The Hong Kong Polytechnic University, Kowloon, Hong Kong SAR, China; ^2^School of Nursing, The Hong Kong Polytechnic University, Kowloon, Hong Kong SAR, China

**Keywords:** social inclusion and exclusion, discrimination, LGBTQ students, mental health, psychological impact

## Abstract

For scholars, practitioners, and legislators concerned about sexual minority adolescents, one of the main goals is to create more positive and inclusive learning environments for this minority group. Numerous factors, such as repeated patterns of homophobic bullying by classmates and others in school, have been a significant barrier to achieving this goal. In addition, lesbian, gay, bisexual, transgender, and queer (LGBTQ) adolescents encounter substantial inequality across a broad spectrum of wellbeing and education consequences. Compared with their heterosexual counterparts, LGBTQ adolescents experience more anxiety, depression, suicidal thoughts, antisocial behavior, poorer academic performance, less school attachment and protection, and a weaker desire to finish their studies. Such discrepancies based on gender and sexuality were linked to more maltreatment encountered by LGBTQ adolescents. It is crucial to recognize the backgrounds and expectations of LGBTQ adolescents to offer them the best resources. To overcome the inequality and obstacles faced by these LGBTQ adolescents, it is essential to examine tools and techniques that can be utilized. This study examined the literature that explains why society fails to provide enough support to LGBTQ students. Specifically, mechanisms explaining how LGBTQ adolescents interact with others in the learning environment and how such discrepancies arise will be examined. Following that, violence and prejudice, which are fundamental causes of psychological problems among LGBTQ adolescents, will be explored. This review paper thus provides supportive strategies for schools to develop more inclusive learning environments for LGBTQ adolescents.

## Introduction

Globally, schools play an essential role in enabling students to acquire college credentials and knowledge, become familiar with the culture, learn about interpersonal relationships, ideals, and standards, and develop survival skills and expertise abilities (Skovdal and Campbell, 2015). When individuals attend schools and colleges and receive a comprehensive education, their chances in life are improved. The community requires their expertise, and they are well equipped to serve it. Given the many roles and advantages of education, school environments need to be protective, stable, inclusive, and pleasant to all students to maximize learning opportunities for everyone to guarantee that school goals are met. Regrettably, colleges and universities worldwide may not be a safe environment for LGBTQ students, who face intimidation, maltreatment, rejection, and other types of discrimination and exploitation (Poynter and Washington, 2005; Fields and Wotipka, 2020; Kurian, 2020). These experiences lead to agony, distress, and anxiety and could have a detrimental effect on LGBTQ students’ physical, psychological and educational wellbeing (Mateo and Williams, 2020; Mallory et al., 2021).

## The Correlations Between Discrimination and Mental Health

There are different forms of discrimination, including verbal abuse, physical aggression, burglaries, accommodation discrimination, and sexual assault (Flores A. R. et al., 2020). Adolescents who identify as LGB experience more severe peer harassment and maltreatment than their straight counterparts (Kolbe, 2020). In the United States, 34% of LGB adolescents experienced bullying at school in the surveyed year, compared to 19% of straight adolescents (Johns et al., 2020). It has been reported that maltreatment of children based on their sexuality has occurred at an early age, as young as eight and nine years old (Evans-Polce et al., 2020).

Proximal minority stressors may negatively affect the lives of LGBTQ individuals. They include internalized homonegativity, societal exclusion expectancies, and the concealment of one’s sexual identity (Delozier et al., 2020). Individuals who have a greater degree of internalized homonegativity express more unfavorable sentiments about themselves due to their sexual orientation (Ocasio et al., 2020). Additionally, LGBTQ people may suffer stress or lack self-confidence due to their sexuality (Minturn et al., 2021). Since sexuality can be concealed from others and that marginalization of LGBTQ people may not be immediately apparent throughout most human relationships (Kachanoff et al., 2020), LGBTQ people need to determine whether, when, how, and to whom they disclose their sexuality (Alonzo and Buttitta, 2019; Lo, 2020). Multiple declarations of their socially marginalized identities might be required, increasing their stress (Daniele et al., 2020). Substantial evidence suggests that bisexual youngsters are at an even greater risk of developing psychological problems than gay/lesbian adolescents (Savin-Williams, 2020), given experiences of stressors associated with “double discrimination” (i.e., exclusion from both the heterosexual and LG populations) and dismissal of one’s self-image as “just a phase” (Ramasamy, 2020).

It should also be noted that LGBTQ students who identify as members of other oppressed groups (for example, racial and cultural minorities, non-Christians, and members of the lower class) may face heightened instances of discrimination in educational institutions. According to The Trevor Project’s 2019 national study on LGBTQ psychological wellness, 71% of LGBTQ youngsters encountered prejudice due to gender and/or sexuality. Additionally, two-thirds of the LGBTQ adolescent interviewees reported that they had been persuaded to alter their sexuality. A survey found that 78% of transgender and non-binary adolescents faced prejudice due to their gender and sexuality, while 70% of LGBTQ adolescents experienced discrimination against their gender expressions. Another study (Platero and López-Sáez, 2020) found that 58% of transgender and non-binary adolescents experienced being discouraged from using the restroom that matched their gender preference.

In addition, study findings indicate that LGBTQ individuals may have serious psychological issues due to their sexuality. A study showed that 39% of LGBTQ interviewees reported actively contemplating suicide in the surveyed year, a majority of them aged between 13 and 17 (Higbee et al., 2020). An astounding 71% of LGBTQ adolescents reported experiencing despair or depression for no fewer than 14 days during the surveyed year (Higbee et al., 2020). While significant progress has been achieved in terms of LGBTQ inclusion over the previous decade, this poll demonstrates that the LGBTQ community, especially younger members, continue to face challenges directed at their sexual identities (Standley, 2020).

## Current Quantitative Study

The effects of loneliness, marginalization, and inequality on psychological health and the assessment of health determinants have been examined in a number of quantitative studies conducted with LGBTQ adolescents (Table 1). The prevalence of suicidal ideation, depression, and drug abuse among LGBTQ adolescents is considerably higher than that of their heterosexual counterparts, highlighting the seriousness and frequency of LGBTQ adolescents’ experiences of inequalities (Price-Feeney et al., 2020). It has been found that LGBTQ adolescents have higher incidences of aggression and victimization as well as more despair and suicidal behavior. These adolescents are also more likely to develop psychosocial disorders, such as alcohol and drug problems and eating disorders (Lannoy et al., 2020). Associations have been established between peer victimization and adverse psychological wellbeing indictors, such as depression, distress, and suicidal tendencies, along with liquor and drug misuse and compromised academic performance, including reduced school involvement and interruptions to academic paths (Brown et al., 2020).

**TABLE 1:** Summarizes the findings of several academic trials and their connection with the mental health impacts on LGBT students in review.

Authors/Studies	Year	Country	Methods	Sample/Participant	Prevalence	Major psychological impact
Grossman et al. (2009)	2009	United States	Grounded theory	*n* = 31 Age: 15–19 Gender: Male (*n* = 19, 61%), Female (*n* = 12, 39%) Sexual orientation: Lesbian (*n* = 6, 19.4%) Bisexual (*n* = 12, 38.7%) Gay (*n* = 8, 25.8%) Male-to-female transgender (*n* = 5, 16.1%)	Two themes generated: 1. Lack of community 2. Lack of empowerment with a concurrent lack of a sense of human agency in school	1. No sense of being apart in school 2. No sense of being a human agency in school
Walls et al. (2019)	2013	United States	Survey	*n* = 7261 Age: 13–21 (Mean age = 16.3) Gender: Male (*n* = 1930, 33.7%), Female (*n* = 3263, 56.9%), Transgender (*n* = 314, 5.5%), Others (*n* = 223, 3.9%) Sexual orientation: Not mentioned	1. Victimization related to sexual orientation & gender expression: Physical harassment was highly correlated with verbal harassment (r = 0.62 for both types) and physical assault (r’s = 0.72 and 0.71, respectively) 2. Structural equation modeling showed that victimization contributed to lower academic outcomes and lower self-esteem	1. Lower academic outcomes 2. Lower self-esteem
Van Bergen et al. (2013)	2013	Netherlands	Survey	*n* = 274 Age (Mean ± SD): 16.77 ± 0.80 Gender: Male (*n* = 106, 38.7%), Female (*n* = 168, 61.3%), Sexual orientation: Not mentioned	1. Suicidal ideation (63.9%) A significant association with victimization at school (Adjusted OR = 1.66, 95% CI = 10.6, 2.6) 2. Suicidal attempt (12.8%) victimization at school (Adjusted OR = 1.98, 95% CI = 1.08–3.62)	1. Suicidal ideation 2. Suicidal attempt
Proulx et al. (2019)	2019	United States	Survey	*n* = 50,072 Age: High school students in Grades 9–12 Gender: Not mentionedSexual orientation: Bisexual (*n* = 3372, 6.7%) Gay/lesbian (*n* = 1259, 2.5%) Heterosexual (*n* = 43331, 86.5%) Not sure (*n* = 2110, 4.3%)	1. Bisexual youth reported the highest frequency of past-year depressive symptoms (62.8%), suicidalthoughts (44.6%), and making a suicide plan (39.3%). 2. Gay/lesbian youth reported the highest frequency of bullying victimizationon school property (34.2%)	1. Depressive symptoms2. Suicidal ideation 3. Suicidal plan 4. Bullying
Walls et al. (2019)	2019	Colorado, United States	Survey	*n* = 9,352 Age: 15.8 (mean) Gender: Male (*n* = 4486, 48%), Female (*n* = 4866, 52%) Sexual orientation: Bisexual (*n* = 704, 7.5%) Gay/Lesbian (*n* = 164, 1.8%) Hetersexual (*n* = 8,161, 87%) Not sure (*n* = 323, 3.7%)	1. Depressive symptoms (*n* = 3,077, 33%) 2. Suicidal attempt (one attempt: *n* = 497, 5.3%; two or more attempts: *n* = 506, 5.4%) 3. School bullying (*n* = 2,087, 22.3%) 4. Online bullying (*n* = 7,655, 18.2%)	1. Depressive symptoms 2. Suicidal attempt 3. Bullying
Wilson and Cariola (2020)	2019	China	Online survey	*n* = 732 Age: 20.3–20.9 Gender: Male (*n* = 512, 69.9%), Female (*n* = 174, 23.8%), Transgender (*n* = 46, 6.3%) Sexual Orientation: Bisexual (*n* = 126, 17.2%) Gay (*n* = 441, 60.2%) Lesbian (*n* = 123, 16.8%) Not sure (*n* = 42, 5.7%)	1. Disagreed or strongly disagreed that LGBTQ students are treated with as much respect as other students (*n* = 234, 32.9%) 2. Suicidal thoughts (*n* = 293, 40%) 3. Depressive symptoms (*n* = 622, 85%)	1. Depressive symptoms 2. Suicidal ideation 3. Not being respected
Hackman et al. (2020)	2020	United States	Qualitative	*n* = 20 Age: 18–25 Gender: Male (*n* = 7, 35%), Female (*n* = 11, 55%), Transgender female (*n* = 2, 10%) Sexual orientation: Bisexual (*n* = 5, 25%) Gay (*n* = 5, 25%) Lesbian (*n* = 3, 15%) Queer (*n* = 3, 15%) Asexual and bisexual (*n* = 1, 5%) Pansexual (*n* = 1, 5%) Homoflexible cupiosexual (*n* = 1, 5%)	Six major themes identified: 1. Interpersonal concerns about disclosure 2. Consequences of sexual assault 3. Hesitance to engage with institutions following sexual assault 4. Sense of LGBTQ+ Community 5. Cisheteronormativity 6. Changes to improve institutional support	1. Feeling of being isolated 2. Negative coping 3. Self-blame
Ybarra et al. (2015)	2015	United States	Online survey	*n* = 5542 Age: 13–18 Gender: Male (*n* = 2260, 40.8%), Female (*n* = 2840, 51.3%), Transgender/gender non-conforming (*n* = 442, 7.9%) Sexual orientation: Bisexual (*n* = 655, 11.8%) Gay, Lesbian, and Queer (*n* = 1282, 23.1%) Questioning, unsure, and others (*n* = 225, 4.1%)Heterosexual (*n* = 3380, 61%)	1. Suicidal thought 39% bisexual; 31% gay, lesbian, 24% questioning/not sure of their sexual identity; 10% heterosexual [p < 0.001] 2. Victims of bullying were five times more likely (OR = 5.61, 95 % CI = 4.11, 7.64) and victims of peer harassment were two times more likely (OR = 2.06, 95 % CI = 1.53, 2.79) than non-victimized youth to report recent suicidal ideation	1. Suicidal ideation 2. Bullying 3. Peer harassment

Quantitative analysis has also centered on defining vulnerability and preventive variables for the psychological wellbeing of LGBTQ adolescents, resulting in the establishment of mitigation, diagnosis, and recovery guidelines, as well as shaping legislation and policy (Lockett, 2020). Family affirmation, for example, provides a protective factor against depression, drug misuse, and suicide among LGBTQ adolescents and young adults. It increases self-esteem, support networks, general wellness, and is a buffer against depression, drug misuse, and suicide (Reyes et al., 2020; Lampis et al., 2021). Thus, household initiatives that inspire and support parents, care providers, and other close relatives have been identified as a beneficial paradigm for preventive strategies. This highlights the strengths and positive impact of constructive parent-child dynamics. Additionally, a recent comprehensive study (Flores A. R. et al., 2020; Flores D. D. et al., 2020) found that elevated degrees of community protection were correlated with a healthy ego while a shortage of community protection was linked with increased levels of stress, anxiety, guilt, alcohol and substance consumption, practices of unsafe sex, and lower levels of self-esteem. McDonald et al. (2021) emphasized the importance of acceptance by family and caregivers and a feeling of connection to a friend/community in LGBTQ youth’s psychological wellbeing.

## Impact on Psychological Wellbeing Among Lesbian, Gay, Bisexual, Transgender, and Queer Students

Mental health entails a positive relationship with people and the pursuit of a productive and fulfilling existence. It has been shown that those who have a high level of mental health tend to be more lighthearted and lead more energetic and pleasant lives (Chan et al., 2021). Owing to their heightened likelihood of experiencing psychological challenges, LGBTQ adolescents are among the most disadvantaged populations in the community (Detrie and Lease, 2007; McGlashan and Fitzpatrick, 2017). According to figures on the LGBTQ community, New Zealand has an estimated 8% of LGB adolescents, the United States has an estimated 7–8% of LGB adolescents (Wilson et al., 2014). According to Aranmolate et al. (2017), LGBTQ adolescents’ psychological health difficulties are associated with a lack of familial recognition and experiences of harassment. They are more likely than their heterosexual counterparts to encounter violent conditions at home and in the larger community, and are exposed to overt and implicit discrimination, violence, vulnerability, and injustice, all of which have a negative effect on psychological wellbeing (Bertrand et al., 2005; Matebeni et al., 2018; Simons and Russell, 2021).

A recent study (Lucassen et al., 2017) found that LGBTQ adolescents were three times more likely than their heterosexual counterparts to exhibit depressive conditions and twice as likely to harm themselves. In the study, 20% of participants attempted suicide, and over half considered it. LGBTQ adolescents were more likely than their non-LGBTQ counterparts to seek therapy in the previous 12 months, at 41.0%. Additionally, the Youth 2000 Survey (Archer et al., 2021) indicates that LGBTQ youth face a higher risk of alcohol or substance usage. In Scotland over the given time, 40% of LGBT adolescents registered as having a psychological disorder, compared to 25% of non-sexual and gender marginalized adolescents and bullying was described as a significant source of anxiety among LGBT participants (Bradbury, 2020; Pachankis et al., 2020).

LGBTQ adolescents, in general, have distinct risk factors, and when such specific threats are associated with common stressful events, this minority group tend to experience increased self-harm, suicidal tendencies, and emotional instability (Eisenberg et al., 2020; Hatchel et al., 2021). These risk factors persist throughout adulthood, with Sexual/Gender Minority (SGM) person 400% more likely to commit suicide and both males and females 150% more likely to experience anxiety, depression, and drug abuse (King et al., 2008; Lothwell et al., 2020). In a 2011 article (Chakraborty et al., 2011), it was found that gay/lesbian individuals experience elevated degrees of psychological discomfort in comparison to straight people.

According to previous studies engaging with minority stress theory (Cyrus, 2017; Fulginiti et al., 2020; Table 1), the rising likelihood of psychological health problems among LGBTQ adolescents is a result of increased social stress, which includes stigma, discrimination, bias, and victimization. Adolescence is a crucial period in cognitive growth, with elevated impact of pressure on psychological wellbeing and an increased susceptibility to substance use (Tavarez, 2020; Fulginiti et al., 2021). At this critical juncture, experiencing discrimination at the hands of academic, clinical, or religious establishments, or internalizing victimization as a consequence of discrimination, transphobia, or biphobia, will create substantial mental difficulties for LGBTQ adolescents (Budge et al., 2020; Formby and Donovan, 2020). Marginalization, loneliness, alienation, bullying, and a lack of supportive grown-ups and spaces all contribute to social tension among LGBTQ adolescents (Grossman et al., 2009; Hafeez et al., 2017).

Stigma establishes individual obstacles for vulnerable adolescents, stopping them from seeking resources (Cortes, 2017). According to Hackman et al. (2020), humiliation, guilt, and apprehension of judgment are all factors explaining why LGBTQ adolescents stop accessing psychological health care. LGBTQ adolescents who are homeless, remote, or drug addicts experience greater obstacles to obtaining assistance (Lucassen et al., 2011, Table 1). If parental or specialist assistance is unavailable, LGBTQ adolescents may seek assistance from peers and resources on online platforms (LaSala, 2015; Pullen Sansfaçon et al., 2020; Town et al., 2021).

Recognition by family members has also been described as a significant factor influencing the psychological wellbeing of LGBTQ adolescents (Afdal and Ilyas, 2020; Buriæ et al., 2020). According to Strauss et al. (2020), familial engagement is represented by openness and sensitivity to the demands of a child. As LGBTQ adolescents feel welcomed and respected, they are more likely to reveal their non-normative identities to family members (Hagai et al., 2020; Endo, 2021). Nevertheless, a huge percentage of LGBTQ adolescents are homeless, indicating that family exclusion is a major risk factor for poor psychological wellbeing (Travers et al., 2020; MacMullin et al., 2021). Durso and Gates (2012) released the findings of a nationwide internet study in the United States and discovered that nearly 68% of their LGBT homeless clients had encountered family abandonment and over 54% had encountered domestic violence.

Adolescence is a transitional stage during which adolescents discover their identity, and for LGBTQ adolescents, it is also the period during which they gain an awareness of their own gender identity and sexual preference (Prock and Kennedy, 2020). Research has shown shifting relationships throughout adolescence and young adulthood, with a corresponding change in commitment to friends and social classes apart from the family, as well as to entities such as education, colleges, religious or political communities (Huang, 2020; Jordan, 2020). Recognition by support communities is a powerful preventive mechanism for LGBTQ children and adolescents (Call et al., 2021). A LGBTQ-friendly climate has a profound impact on their psychological development and well-being. Perceptions of social integration with grown-ups help LGBTQ adolescents overcome challenges, especially during the precarious developmental phase when they are developing their sense of self (Proulx et al., 2019).

## The Supportive Strategies for Inclusive Schools

The interaction between a person and his or her environment affects personal growth and development according to the social ecological model. The risk of suicidal behaviors among LGBTQ adolescents is influenced by a number of contextual factors including schools. Institute of Medicine asked for further research in 2011, focusing specifically on the impact of protective school policies and students’ perceptions of their school environments on their health and well-being (Ancheta et al., 2021). Much research pointed out that schools are well-positioned to address health disparities by creating safe and supportive school climates for LGBTQ youth (Gower et al., 2018; Woodford et al., 2018; Table 2). Evidence shows that a safe and supportive climate is related to lower odds of student bullying involvement, some types of risky alcohol use and drug use, and victimization. A safe climate event may reduce LGBTQ adolescents’ risk of suicidal thoughts (Konishi et al., 2013; Kosciw et al., 2013; Hatzenbuehler et al., 2014; Gower et al., 2018). Having a supportive school environment and a sense of belonging to school were associated with lower levels of minority stress and better academic results, health, and wellbeing among LGBTQ students (Denny et al., 2016; Perales and Campbell, 2020). Research has suggested multiple strategies for school-based support, including policies, supporting LGBTQ students organizations, educator intervention and LGBTQ related curriculum (Konishi et al., 2013; Kosciw et al., 2013).

**TABLE 2:** The supportive strategies/services and implication with inclusion studies in review.

Author/Study	Country	Supportive strategies/Service	Sample/Participants	Study Findings	Implication
Gower et al. (2018)	United States	Student-focused (e.g., GSA), staff-focused (e.g., professional development), and a combination (e.g., point person for LGBT student issues). These programs include some elements of professional development, classroom activities, more formal curriculum, and school-wide communication of inclusive norms through stickers and posters.	Student-level data: 8th (*n* = 121), 9th (*n* = 121), or 11th (*n* = 119) grades (*n* = 176 schools in total) completing the 2013 Minnesota Student Survey (MSS). Assuming a 5% LGBQ rate. School-level data: *N* = 31,183 students in 103 schools.	This study provides promising evidence that school efforts to promote safe and supportive climates for LGBQ youth through multiple practices are associated with lower odds of student bullying involvement	Findings support school-wide efforts to create supportive climates for LGBQ youth as part of a larger bullying prevention strategy
Kosciw et al. (2013)	United States	Safe school policies, supportive school personnel, and gay–straight alliance (GSA) clubs	*N* = 5,7630 lesbian, gay, bisexual, and transgender students between the ages of 13 and 21 (*M* = 16.3 years)	School-based supports contributed to lower victimization and better academic outcomes	A hostile school climate has serious ramifications for LGBT students but institutional supports can play a significant role in making schools safer for these students
Woodford et al. (2018)	United States	Using SEM, indicate that antidiscrimination policies that enumerate both sexual orientation and gender identity (vs. only sexual orientation), offering at least one for-credit course on LGBTQ topics, and the ratio of LGBTQ student organizations to the student body size	*N* = 268, 58% undergraduates; 25% students of color; 62% gay/lesbian from 58 colleges completed an anonymous online survey addressing experiential heterosexism and psychological well-being	Colleges can work to decrease heterosexist discrimination on campus by utilizing multiple strategies: policies, formal educational resources, and by supporting LGBTQ student organizations	The results underscore the importance of particular structural initiatives on campus in protecting LGBQ+ collegians from discrimination and highlight the value of studying specific structural initiatives when investigating structural stigma and inclusion
Willging et al. (2016)	United States	“RLAS” (Implementing School Nursing Strategies to Reduce LGBTQ Adolescent Suicide), builds on the Exploration, Preparation, Implementation, and Sustainment (EPIS) conceptual framework and the Dynamic Adaptation Process (DAP) to implement EB strategies in U.S. high schools	Compared the LGBTQ students and their peers in RLAS intervention schools (*n* = 20) with those in usual care schools (*n* = 20)	The conceptual framework and methods for this novel nurse-led intervention are applicable to addressing LGBTQ youth suicide and the health-related concerns of other pediatric populations in schools as well	Through its collaborative processes to refine, improve, and sustain EB strategies in these systems, the RLAS represents an innovative contribution to implementation science that also addresses a pressing public health challenge
Konishi et al. (2013)	Canada	Gay-straight alliances and anti-homophobic bullying policies	A population-based sample of students in grades 8 through 12 from the British Columbia Adolescent Health Survey of 2008 (*N* = 21,708)	Gay-straight alliances and anti-homophobic bullying policies were linked to significantly lower odds of some but not all types of recent risky alcohol use and past-year harms from alcohol or drug use, but almost exclusively in schools where the policies or gay-straight alliances had been established for at least 3 years; and among lesbian, gay and bisexual adolescents, only for girls	Our findings suggest that these school-based strategies (gay-straight alliances and anti-homophobia policies) to reduce homophobia and foster school inclusion may be beneficial in reducing problem alcohol use among all students, not just sexual minority students
Heck et al. (2014)	United States	GSAs	*N* = 475, LGBT high school students (*M*age = 16.79) who completed an online survey	LGBT youth attending a high school without a GSA evidenced increased risk for using illicit drugs and prescription drug misuse. GSAs help foster school environments where the burden of minority stressors is reduced	The importance of providing LGBT youth with opportunities for socialization and support within the school setting
Day et al. (2019)	United States	Sexual orientation and gender identity (SOGI) policy	2013–2015 California Healthy Kids Survey (*n* = 113, 148)	The number of SOGI-focused policies in schools was associated with less victimization and SOG-based bullying for LGB youth and with higher grades for transgender youth. A greater number of SOGI-focused policies was associated with lower truancy for all students. The policies operate differentially for LGB and transgender youth, though are associated with positive school experiences for both	A “one size fits all” approach to school policy may support LGB youth more than transgender youth. Policies are directly responsive to the unique experiences and needs of transgender youth may be necessary to reduce these disparities
Tollemache et al. (2021)	United Kingdom	LGBT teaching within the undergraduate curricula of United Kingdom medical schools	37 United Kingdom Medical Schools with students currently enrolled in a primary undergraduate medical training course were asked between December 2019–March 2020 to complete a cross-sectional online survey comprised of 30 questions	A significant variation in the amount and breadth of content within the undergraduate curricula of United Kingdom medical schools, which is a good degree of coverage in topics that serve to address the areas identified by Stonewall as being important to LGBT patients	The study provides suggestions for undergraduate curriculum development leads about how to improve the level and range of LGBT-associated content in their course
Ybarra et al. (2015)	Canada	LGBTQ-inclusive education strategies	They present quantitative and qualitative results of a national survey of more than 3,700 Canadian high school students undertaken in order to investigate what life is like at school for sexual and gender minority students	The findings show that even modest efforts to shift the balance of heteronormative discourse on behalf of LGBTQ students can have profound effects on the experiences and perceptions of sexual and gender minority youth, which we argue would go a long way in reducing incidents of suicidality among LGBTQ youth. In many jurisdictions across Canada, LGBTQ-inclusive policies have attempted to improve school climates and reduce the effects of homophobia and transphobia in schools	These initiatives, along with the work done by Eagle Canada to create a National Youth Suicide Prevention Strategy, are important steps in addressing the needs of LGBTQ youth
Kull et al. (2016)	United States	Antibullying policies that explicitly prohibit bullying based upon a student’s sexual orientation, gender identity, and/or gender expression (SOGIE; i.e., SOGIE-inclusive policies)	Data from a national survey of LGBT students’ school experiences (7,040 LGBT students from 2,952 unique school districts)	LGBT students in districts with SOGIE protections in their policies reported greater school safety, less victimization based on their sexual orientation and gender expression, and less social aggression than students with generic policies or no/unidentified policies	Antibullying policies explicitly enumerating SOGIE protections can improve LGBT school experiences and that generic policies may not sufficiently protect LGBT students from bullying and harassment
Proulx et al. (2019)	United States	Gay-straight alliances (GSAs)	*N* = 62,923 participants in 15 primary studies	GSA presence is associated with significantly lower levels of youth’s self-reports of homophobic victimization, fear for safety, and hearing homophobic remarks	The findings of this meta-analysis provide evidence to support GSAs as a means of protecting LGTBQ+ youth from school-based victimization
Lessard et al. (2020)	United States	Gay-straight alliances (GSAs, also referred to as gender-sexuality alliances)	A sample of diverse sexual and gender minority adolescents (*N* = 17,112; *M*age = 15.57)	Lower levels of multiple forms of bias-based bullying (based on body weight, gender, religion, disability, gender typicality, and sexuality) at schools with versus without GSAs, and in turn higher perceived school safety, as well as higher grades and reduced school suspension (due to less weight- and sexuality-based bullying)	The findings shed light on the broad-reaching stigma-reduction potential of GSAs
Hackman et al. (2020)	United States	Curriculum and a Gay-Straight Alliance	*N* = 1415 students in 28 high schools in California from the Preventing School Harassment (PSH) Survey	When schools included lesbian, gay, bisexual, transgender, and queer (LGBTQ) issues in the curriculum and had a Gay-Straight Alliance, students perceived their schools as safer for gender nonconforming male peers	The findings suggest that school administrators, teachers, and other school personnel who implement safe schools policies and practices need to be intentionally inclusive to the needs of gender nonconforming students
Hatzenbuehler et al. (2014)	United States	School climates	Data on sexual orientation and past-year suicidal thoughts, plans, and attempts were from the pooled 2005 and 2007 Youth Risk Behavior Surveillance Surveys from 8 states and cities.	School climates that protect sexual minority students may reduce their risk of suicidal thoughts	
Denny et al. (2016)	New Zealand	Supportive school environments	A nationally representative sample of students (*N* = 9,056) and teachers (*N* = 2,901) from 96 high schools in 2007	Teacher reports of more supportive school environments for GLBT students were associated with fewer depressive symptoms among male sexual minority students but not for female sexual minority students. Students reported a more supportive school environment, male sexual minority students reported fewer depressive symptoms, and less suicidality than in schools where students reported less favorable school climates	Schools play an important role in providing safe and supportive environments for male sexual minority students
Van Bergen et al. (2013)	Australia	School belonging	Data from an Australian national probability sample of 14–15-year olds (Longitudinal Study of Australian Children, *n* = 3204)	The support and belonging variables were responsible for 49–70% of the associations between sexual minority status and the health/well-being outcomes, with school belonging being the most important mediator	These findings have important implications for health equity policy and practice

More inclusive policies could contribute to the school climate at the macro level. These policies include antidiscrimination policies (Woodford et al., 2018) and anti-homophobic bullying policies (Konishi et al., 2013). Compared with students at schools with generic policies or no/unidentified policies, LGBTQ students in districts with sexual orientation, gender identity, and/or gender expression (SOGIE) protections in their policies reported greater school safety, less victimization based on their sexual orientation and gender expression, and less social aggression (Kull et al., 2016). Moreover, a greater number of SOGIE-focused policies was associated with lower truancy for all students (Day et al., 2019).

Furthermore, gay–straight alliance (GSAs) has been one of the major sources of support in high schools in the United States and Canada. GSAs are student-led, school-based clubs that aim to provide a safe environment in the school context for LGBTQ students, as well as their straight allies (Toomey et al., 2011. In recent decades, the number of GSAs in schools has increased dramatically, with over 4,000 GSAs registered in the United States (Toomey et al., 2011). Research has suggested that a high school with a GSA can decrease LGBTQ students-risks for using illicit drugs and prescription drug misuse and reduce their burden of minority stressors (Heck et al., 2014). GSAs foster inclusive school environments not only for LGBT + students but for all students, thereby contributing to lower levels of homonegative victimization, fear for safety, homophobic remarks and multiple forms of bias-based bullying (based on body weight, gender, religion, disability, gender typicality, sexuality) (Marx and Kettrey, 2016; Lessard et al., 2020).

Educator intervention and LGBTQ related curriculum also constitute prevention strategies of inclusive schools. Teachers and school staff—in particular, the medical staff—could be provided with LGBTQ sensitive training and LGBTQ medical curricula, which are important for supportive climate building and LGBTQ students’ wellbeing (Gower et al., 2018; Tollemache et al., 2021). By strengthening teachers’ analytical awareness of alienation experienced by children and adolescents, teachers may flourish in school, promoting equal opportunity principles and teaching students about love and consideration, justice and freedom (Glazzard and Stones, 2020). Willging et al. (2016) demonstrated that RLAS (Implementing School Nursing Strategies to Reduce LGBTQ Adolescent Suicide) is applicable to novel nurse-led intervention to address LGBTQ youth suicide and health-related concerns of other students. All these strategies have shown the importance of structural initiatives on campus in protecting LGBTQ students from discrimination (Woodford et al., 2018).

## Discussion

Around the world, the importance of an inclusive school climate to LGBTQ student has been suggested and advocated for reducing the risk of violence and discrimination and enhancing their psychological wellbeing. However, most quantitative studies were concentrated in the Northern America, particularly the United States. There remains a lack of research about LGBTQ students’ wellbeing in developing countries. Moreover, much research used the data from health or psychological surveys to state that policies, GSAs club, educator intervention, and LGBTQ related curriculum could improve the school climate; nevertheless, less experimental research could provide evidence and specific methods to guide schools. Further, due to the disparities among LGBTQ students, a ‘one size fits all’ approach to school policy might not fit all LGBTQ students. Day et al. (2019) have demonstrated that SOGIE-focused policy may support LGBTQ youth more than transgender youth.

Toomey et al. (2011) have pointed out that students perceived their schools as safer for gender nonconforming male peers when schools included LGBTQ issues in the curriculum and had GSAs. Given the growing significance of LGBTQ people as active, respected, and noticeable members of society (Chan, 2021b), it is critical to promote LGBTQ acceptance within and beyond campus (Stones and Glazzard, 2020). LGBTQ students are more likely to report negative school performance when confronted with significant obstacles such as bullying, assault, and a lack of role models. Schools should uphold diversity, decency, compassion, and consideration (Chan, 2021c). Additionally, deans of medical schools have suggested to increase teaching materials related to LGBTQ issues in order to improve medical services in schools (Van Bergen et al., 2013).

This article has collected and analyzed the existing literature to indicate violence and prejudice as fundamental causes of psychological problems among LGBTQ adolescents and identify supportive strategies for schools to build a LGBTQ-friendly environment. However, it is limited because previous studies still primarily focus on developed countries and offer limited insights into possible interventions in different contexts. This study has suggested how treatments should be further developed to guarantee lasting welfare and inclusion of LGBTQ adolescents.

## Conclusion

As LGBTQ individuals are becoming a more dedicated, respected, and observable component of humanity (Chan, 2021a), schools play a crucial part in ensuring that all children and adolescents realize that prejudice and discrimination are unacceptable. By teaching young people about all kinds of discrimination and their negative impact, critical pedagogy plays a crucial part in advancing human rights. It inspires optimism for the potential creation of a more just and fair society and empowers young people to be ethical new generations (Glazzard and Stones, 2021). Mental health problems faced by LGBTQ youth are largely associated with discrimination, prejudice, and a lack of support from family, schools, and society at large. Increasing levels of support and acceptance for LGBTQ youth will most likely require political and social change in today’s world, such as legalizing same-sex marriage and liberalizing cultural norms. Future research should continue to attend to LGBTQ students’ health and educational needs and identify possible interventions in order to enhance their wellbeing.

## Author Contributions

AC and DW carried out the outline of this manuscript. AC wrote the manuscript with support from JH and IL. EY and IL gave valuable comments and suggestion. EY helped to supervise the whole manuscript. All authors contributed to the article and approved the submitted version.

## Conflict of Interest

The authors declare that the research was conducted in the absence of any commercial or financial relationships that could be construed as a potential conflict of interest.

## Publisher’s Note

All claims expressed in this article are solely those of the authors and do not necessarily represent those of their affiliated organizations, or those of the publisher, the editors and the reviewers. Any product that may be evaluated in this article, or claim that may be made by its manufacturer, is not guaranteed or endorsed by the publisher.

## References

[B1] Afdal A. Ilyas A. (2020). How psychological well-being of adolescent based on demography indicators? *JPPI* 6 53–68.

[B2] Alonzo D. J. Buttitta D. J. (2019). Is “coming out” still relevant? Social justice implications for LGB-membered families. *J. Fam. Theory Rev*. 11, 354–366.

[B3] Ancheta A. J. Bruzzese J. M. Hughes T. L. (2021). The Impact of Positive School Climate on Suicidality and Mental Health Among LGBTQ Adolescents: A Systematic Review. *J. Sch. Nurs.* 37 75–86. 10.1177/1059840520970847 33287652 PMC8142116

[B4] Aranmolate R. Bogan D. R. Hoard T. Mawson A. R. (2017). Suicide Risk Factors among LGBTQ Youth: Review. *JSM Schizophr.* 2:1011.

[B5] Archer D. Clark T. C. Lewycka S. DaRocha M. Fleming T. (2021). *Youth19 Rangatahi Smart Survey, Data Dictionary (Edited from The Adolescent Health Research Group Previous Youth2000 series Data Dictionaries).* Wellington: The University of Auckland and Victoria University of Wellington.

[B6] Bertrand M. Chugh D. Mullainathan S. (2005). Implicit discrimination. *Am. Econom. Rev.* 95 94–98.

[B7] Bradbury A. (2020). Mental health stigma: The impact of age and gender on attitudes. *Commun. Mental Health J.* 56 933–938. 10.1007/s10597-020-00559-x 32002711

[B8] Brown C. Porta C. M. Eisenberg M. E. McMorris B. J. Sieving R. E. (2020). Family Relationships and the Health and Well-Being of Transgender and Gender-Diverse Youth: A Critical Review. *LGBT Health* 7 407–419. 10.1089/lgbt.2019.0200 33170062

[B9] Budge S. L. Domínguez S. Jr. Goldberg A. E. (2020). Minority stress in nonbinary students in higher education: The role of campus climate and belongingness. *Psychol. Sexual Orient. Gender Divers.* 7:222.

[B10] Buriæ J. Garcia J. R. Štulhofer A. (2020). Is sexting bad for adolescent girls’ psychological well-being? A longitudinal assessment in middle to late adolescence. *New Med. Soc.* 2020:1461444820931091.

[B11] Call D. C. Challa M. Telingator C. J. (2021). Providing Affirmative Care to Transgender and Gender Diverse Youth: Disparities, Interventions, and Outcomes. *Curr. Psychiatry Rep.* 23 1–10. 10.1007/s11920-021-01245-9 33851310

[B12] Chakraborty A. McManus S. Brugha T. S. Bebbington P. King M. (2011). Mental health of the non-heterosexual population of England. *Br. J. Psychiatry* 198 143–148. 10.1192/bjp.bp.110.082271 21282785

[B13] Chan A. S. W. (2021b). Book Review: Safe Is Not Enough: Better Schools for LGBTQ Students (Youth Development and Education Series). *Front. Psychol.* 12:704995. 10.3389/fpsyg.2021.704995

[B14] Chan A. S. W. (2021c). Book Review: The Educator’s Guide to LGBT+ Inclusion: A Practical Resource for K-12 Teachers, Administrators, and School Support Staff. *Front. Psychol.* 12:692343. 10.3389/fpsyg.2021.692343

[B15] Chan A. S. W. (2021a). Book Review: The Gay Revolution: The Story of the Struggle. *Front. Psychol.* 12:677734. 10.3389/fpsyg.2021.677734

[B16] Chan A. S. W. Ho J. M. C. Li J. S. F. Tam H. L. Tang P. M. K. (2021). Impacts of COVID-19 Pandemic on Psychological Well-Being of Older Chronic Kidney Disease Patients. *Front. Med.* 8:666973. 10.3389/fmed.2021.666973 34124096 PMC8187602

[B17] Cortes R. N. (2017). Stigma and discrimination experiences in health care settings more evident among transgender people than males having sex with males (MSM) in Indonesia, Malaysia, The Philippines and Timor leste: key results. *Sex. Transm. Infect*. 93:A202. 10.1136/sextrans-2017-053264.525

[B18] Cyrus K. (2017). Multiple minorities as multiply marginalized: Applying the minority stress theory to LGBTQ people of color. *J. Gay Lesbian Mental Health* 21 194–202. 10.1080/19359705.2017.1320739

[B19] Daniele M. Fasoli F. Antonio R. Sulpizio S. Maass A. (2020). Gay voice: Stable marker of sexual orientation or flexible communication device? *Arch. Sexual Behav.* 49 2585–2600. 10.1007/s10508-020-01771-2 32617773

[B20] Day J. K. Ioverno S. Russell S. T. (2019). Safe and supportive schools for LGBT youth: Addressing educational inequities through inclusive policies and practices. *J. School Psychol.* 74 29–43. 10.1016/j.jsp.2019.05.007 31213230 PMC10409610

[B21] Delozier A. M. Kamody R. C. Rodgers S. Chen D. (2020). Health disparities in transgender and gender expansive adolescents: A topical review from a minority stress framework. *J. Pediatric Psychol.* 45 842–847. 10.1093/jpepsy/jsaa040 32626901

[B22] Denny S. Lucassen M. F. G. Stuart J. Fleming T. Bullen P. Peiris-John R. (2016). The Association Between Supportive High School Environments and Depressive Symptoms and Suicidality Among Sexual Minority Students. *J. Clin. Child Adolesc. Psychol.* 45 248–261. 10.1080/15374416.2014.958842 25469988

[B23] Detrie P. M. Lease S. H. (2007). The relation of social support, connectedness, and collective self-esteem to the psychological well-being of lesbian, gay, and bisexual youth. *J. Homosex.* 53 173–199. 10.1080/00918360802103449 18689197

[B24] Durso L. E. Gates G. J. (2012). *Serving our youth: Findings from a national survey of services providers working with lesbian, gay, bisexual and transgender youth who are homeless or at risk of becoming homeless.* Los Angeles, CA: The Williams Institute with True Colors Fund and The Palette Fund.

[B25] Eisenberg M. E. Gower A. L. Watson R. J. Porta C. M. Saewyc E. M. (2020). LGBTQ Youth-Serving Organizations: What Do They Offer and Do They Protect Against Emotional Distress? *Ann. LGBTQ Public Populat. Health* 1 63–79. 10.1891/lgbtq.2019-0008 11261958

[B26] Endo R. (2021). Diversity, equity, and inclusion for some but not all: LGBQ Asian American youth experiences at an urban public high school. *Multicult. Educat. Rev.* 13 25–42. 10.1080/2005615x.2021.1890311

[B27] Evans-Polce R. J. Veliz P. T. Boyd C. J. Hughes T. L. McCabe S. E. (2020). Associations between sexual orientation discrimination and substance use disorders: differences by age in US adults. *Soc. Psychiatry Psychiatric Epidemiol.* 55 101–110. 10.1007/s00127-019-01694-x 30903234 PMC6755065

[B28] Fields X. Wotipka C. M. (2020). Effect of LGBT anti-discrimination laws on school climate and outcomes for lesbian, gay, and bisexual high school students. *J. LGBT Youth* 2020 1–23. 10.1080/19361653.2020.1821276

[B29] Flores A. R. Langton L. Meyer I. H. Romero A. P. (2020). Victimization rates and traits of sexual and gender minorities in the United States: Results from the National Crime Victimization Survey, 2017. *Sci. Adv.* 6:eaba6910. 10.1126/sciadv.aba6910 33008905 PMC7852385

[B30] Flores D. D. Meanley S. P. Bond K. T. Agenor M. Relf M. V. Barroso J. V. (2020). Topics for Inclusive Parent-Child Sex Communication by Gay, Bisexual, Queer Youth. *Behav. Med.* 2020 1–10. 10.1080/08964289.2019.1700481 32027581 PMC7416429

[B31] Formby E. Donovan C. (2020). Sex and relationships education for LGBT+ young people: lessons from UK youth work. *Sexualities* 23, 1155–1178. 10.1177/1363460719888432

[B32] Fulginiti A. Goldbach J. T. Mamey M. R. Rusow J. Srivastava A. Rhoades H. (2020). Integrating minority stress theory and the interpersonal theory of suicide among sexual minority youth who engage crisis services. *Suicide Life Threat. Behav.* 50, 601–616. 10.1111/sltb.12623 32048340

[B33] Fulginiti A. Rhoades H. Mamey M. R. Klemmer C. Srivastava A. Weskamp G. (2021). Sexual minority stress, mental health symptoms, and suicidality among LGBTQ youth accessing crisis services. *J. Youth Adolesc.* 50 893–905. 10.1007/s10964-020-01354-3 33206318

[B34] Glazzard J. Stones S. (2020). Supporting student teachers with minority identities: The importance of pastoral care and social justice in initial teacher education. *Res. Informed Teacher Learn.* 2020 127–138.

[B35] Glazzard J. Stones S. (2021). Running Scared? A Critical Analysis of LGBTQ+ Inclusion Policy in Schools. *Front. Sociol.* 6:613283. 10.3389/fsoc.2021.613283 34179181 PMC8220066

[B36] Gower A. L. Forster M. Gloppen K. Johnson A. Z. Eisenberg M. E. Connett J. E. (2018). School practices to foster LGBT-supportive climate: Associations with adolescent bullying involvement. *Prevent. Sci.* 19 813–821. 10.1007/s11121-017-0847-4 29032496

[B37] Grossman A. H. Haney A. P. Edwards P. Alessi E. J. Ardon M. Howell T. J. (2009). Lesbian, Gay, Bisexual and Transgender Youth Talk about Experiencing and Coping with School Violence: A Qualitative Study. *J. LGBT Youth* 6 24–46. 10.1080/19361650802379748

[B38] Hackman C. L. Bettergarcia J. N. Wedell E. Simmons A. (2020). Qualitative exploration of perceptions of sexual assault and associated consequences among LGBTQ+ college students. *Psychol. Sex. Orient. Gender Divers.* 2020:sgd0000457. 10.1037/sgd0000457

[B39] Hafeez H. Zeshan M. Tahir M. A. Jahan N. Naveed S. (2017). Health care disparities among lesbian, gay, bisexual, and transgender youth: a literature review. *Cureus* 9:e1184. 10.7759/cureus.1184 28638747 PMC5478215

[B40] Hagai E. B. Annechino R. Young N. Antin T. (2020). Intersecting sexual identities, oppressions, and social justice work: Comparing LGBTQ Baby Boomers to Millennials who came of age after the 1980s AIDS epidemic. *J. Soc. Iss.* 76 971–992. 10.1111/josi.12405 34565893 PMC8459889

[B41] Hatchel T. Torgal C. El Sheikh A. J. Robinson L. E. Valido A. Espelage D. L. (2021). LGBTQ youth and digital media: online risks. *Child Adolesc. Online Risk Expos.* 2021 303–325. 10.1016/j.jadohealth.2016.11.023 28108088

[B42] Hatzenbuehler M. L. Birkett M. Van Wagenen A. Meyer I. H. (2014). Protective school climates and reduced risk for suicide ideation in sexual minority youths. *Am. J. Public Health* 104 279–286. 10.2105/AJPH.2013.301508 24328634 PMC3935661

[B43] Heck N. C. Livingston N. A. Flentje A. Oost K. Stewart B. T. Cochran B. N. (2014). Reducing risk for illicit drug use and prescription drug misuse: High school gay-straight alliances and lesbian, gay, bisexual, and transgender youth. *Addict. Behav.* 39 824–828. 10.1016/j.addbeh.2014.01.007 24531638 PMC4066611

[B44] Higbee M. Wright E. R. Roemerman R. M. (2020). Conversion Therapy in the Southern United States: Prevalence and Experiences of the Survivors. *J. Homosex.* 2020 1–20. 10.1080/00918369.2020.1840213 33206024

[B45] Huang Y. T. (2020). Lesbian, Gay, and Bisexual (LGB) Young Adults’ Relational Well-Being Before and After Taiwanese Legalization of Same-Sex Marriage: A Qualitative Study Protocol. *Int. J. Qualitat. Methods* 19:1609406920933398.

[B46] Johns M. M. Lowry R. Haderxhanaj L. T. Rasberry C. N. Robin L. Scales L. (2020). Trends in violence victimization and suicide risk by sexual identity among high school students—Youth Risk Behavior Survey, United States, 2015–2019. *MMWR Suppl.* 69:19. 10.15585/mmwr.su6901a3 32817596 PMC7440203

[B47] Jordan F. (2020). Changing the Narrative for LGBTQ Adolescents: A Literature Review and Call for Research into Narrative Therapy to Improve Family Acceptance of LGBTQ Teens. *Counsel. Fam. Therapy Scholar. Rev.* 3:6.

[B48] Kachanoff F. J. Cooligan F. Caouette J. Wohl M. J. (2020). Free to fly the rainbow flag: the relation between collective autonomy and psychological well-being amongst LGBTQ+ individuals. *Self Ident.* 2020 1–33.

[B49] Khanlou N. Koh J. G. Mill C. (2008). Cultural Identity and Experiences of Prejudice and Discrimination of Afghan and Iranian Immigrant Youth. *Int. J. Ment. Health Addict.* 6 494–513. 10.1007/s11469-008-9151-7

[B50] King M. Semlyen J. Tai S. S. Killaspy H. Osborn D. Popelyuk D. (2008). A systematic review of mental disorder, suicide, and deliberate self harm in lesbian, gay and bisexual people. *BMC Psychiatry* 8:70. 10.1186/1471-244X-8-70 18706118 PMC2533652

[B51] Kolbe S. M. (2020). Creating Safety in Schools for LGBT and Gender Non-Conforming Students. *BU J. Graduate Stud. Educat.* 12 17–21.

[B52] Konishi C. Saewyc E. Homma Y. Poon C. (2013). Population-level evaluation of school-based interventions to prevent problem substance use among gay, lesbian and bisexual adolescents in Canada. *Prevent. Med.* 57 929–933. 10.1016/j.ypmed.2013.06.031 23850517 PMC4709168

[B53] Kosciw J. G. Palmer N. A. Kull R. M. Greytak E. A. (2013). The Effect of Negative School Climate on Academic Outcomes for LGBT Youth and the Role of In-School Supports. *J. School Viol.* 12 45–63. 10.1080/15388220.2012.732546

[B54] Kull R. M. Greytak E. A. Kosciw J. G. Villenas C. (2016). Effectiveness of school district antibullying policies in improving LGBT youths’ school climate. *Psychol. Sex. Orient. Gender Divers.* 3:407.

[B55] Kurian N. (2020). Rights-protectors or rights-violators? Deconstructing teacher discrimination against LGBT students in England and the UN Convention on the Rights of the Child as an advocacy tool. *Int. J. Hum. Rights* 24 1080–1102.

[B56] Lampis J. De Simone S. Belous C. K. (2021). Relationship satisfaction, social support, and psychological well-being in a sample of Italian lesbian and gay individuals. *J. GLBT Fam. Stud.* 17:49–62.

[B57] Lannoy S. Mange J. Leconte P. Ritz L. Gierski F. Maurage P. (2020). Distinct psychological profiles among college students with substance use: A cluster analytic approach. *Addict. Behav.* 109:106477. 10.1016/j.addbeh.2020.106477 32485549

[B58] LaSala M. C. (2015). Condoms and connection: Parents, gay and bisexual youth, and HIV risk. *J. Marital Fam. Therapy* 41 451–464. 10.1111/jmft.12088 25099281

[B59] Lessard L. M. Watson R. J. Puhl R. M. (2020). Bias-based bullying and school adjustment among sexual and gender minority adolescents: the role of gay-straight alliances. *J. Youth Adolesc.* 49 1094–1109. 32246306 10.1007/s10964-020-01205-1

[B60] Lockett G. (2020). *Spiritual, Cultural, and Religious Influences on the Psychological Well-being of LGBTQ Individuals.* Ph. D. Thesis. Tennessee: Tennessee State University.

[B61] Lo I. P. Y. (2020). Family formation among lalas (lesbians) in urban China: strategies for forming families and navigating relationships with families of origin. *J. Sociol*. 56, 629–645.

[B62] Lothwell L. E. Libby N. Adelson S. L. (2020). Mental Health Care for LGBT Youths. *Focus* 18 268–276. 10.1176/appi.focus.20200018 33162863 PMC7587912

[B63] Lucassen M. F. G. Stasiak K. Samra R. Frampton C. M. A. Merry S. N. (2017). Sexual minority youth and depressive symptoms or depressive disorder: A systematic review and meta-analysis of population-based studies. *Austral. N Z. J. Psychiatry* 51 774–787. 10.1177/0004867417713664 28565925

[B64] Lucassen M. F. Merry S. N. Robinson E. M. Denny S. Clark T. Ameratunga S. (2011). Sexual attraction, depression, self-harm, suicidality and help-seeking behaviour in New Zealand secondary school students. *Austral. N Z. J. Psychiatry* 45 376–383. 10.3109/00048674.2011.559635 21361850

[B65] MacMullin L. N. Bokeloh L. M. Nabbijohn A. N. Santarossa A. van der Miesen A. I. Peragine D. E. (2021). Examining the Relation Between Gender Nonconformity and Psychological Well-Being in Children: The Roles of Peers and Parents. *Arch. Sex. Behav.* 50 823–841. 10.1007/s10508-020-01832-6 33185827

[B66] Mallory C. Vasquez L. A. Brown T. N. Momen R. E. Sears B. (2021). *The Impact of Stigma and Discrimination Against LGBT People in West Virginia.* Los Angeles, CA: Williams Institute.

[B67] Marx R. A. Kettrey H. H. (2016). Gay-straight alliances are associated with lower levels of school-based victimization of LGBTQ+ youth: A systematic review and meta-analysis. *J. Youth Adolesc.* 45 1269–1282. 10.1007/s10964-016-0501-7 27221632

[B68] Matebeni Z. Monro S. Reddy V. (eds) (2018). *Queer in Africa: LGBTQI Identities, Citizenship, and Activism.* London: Routledge.

[B69] Mateo C. M. Williams D. R. (2020). Addressing bias and reducing discrimination: The professional responsibility of health care providers. *Acad. Med.* 95 S5–S10. 10.1097/ACM.0000000000003683 32889919

[B70] McDonald S. E. Murphy J. L. Tomlinson C. A. Matijczak A. Applebaum J. W. Wike T. L. (2021). Relations between sexual and gender minority stress, personal hardiness, and psychological stress in emerging adulthood: Examining indirect effects via human-animal interaction. *Youth Soc.* 2021:0044118X21990044.

[B71] McGlashan H. Fitzpatrick K. (2017). LGBTQ youth activism and school: Challenging sexuality and gender norms. *Health Educat.* 117 485–497. 10.1108/he-10-2016-0053

[B72] Minturn M. S. Martinez E. I. Le T. Nokoff N. Fitch L. Little C. E. (2021). Early Intervention for LGBTQ Health: A 10-Hour Curriculum for Preclinical Health Professions Students. *MedEdPORTAL* 17:11072. 10.15766/mep_2374-8265.11072 33473382 PMC7809930

[B73] Ocasio M. A. Tapia G. R. Lozano A. Carrico A. W. Prado G. (2020). Internalizing symptoms and externalizing behaviors in Latinx adolescents with same sex behaviors in Miami. *J. LGBT Youth* 2020 1–17. 10.1080/19361653.2020.1777245 PMC9562298 36247028

[B74] Pachankis J. E. Clark K. A. Burton C. L. Hughto J. M. W. Bränström R. Keene D. E. (2020). Sex, status, competition, and exclusion: Intraminority stress from within the gay community and gay and bisexual men’s mental health. *J. Pers. Soc. Psychol.* 119:282. 10.1037/pspp0000282 31928026 PMC7354883

[B75] Perales F. Campbell A. (2020). Health Disparities Between Sexual Minority and Different-Sex-Attracted Adolescents: Quantifying the Intervening Role of Social Support and School Belonging. *LGBT Health* 7 146–154. 10.1089/lgbt.2019.0285 32155106

[B76] Platero R. L. López-Sáez M. Á (2020). Support, cohabitation and burden perception correlations among LGBTQA+ youth in Spain in times of COVID-19. *J. Children’s Serv.* 15 221–228. 10.1108/jcs-07-2020-0037

[B77] Poynter K. J. Washington J. (2005). Multiple identities: Creating community on campus for LGBT students. *New Direct. Stud. Serv.* 2005 41–47. 10.1002/ss.172

[B78] Price-Feeney M. Green A. E. Dorison S. (2020). Understanding the mental health of transgender and nonbinary youth. *J. Adolesc. Health* 66 684–690. 10.1016/j.jadohealth.2019.11.314 31992489

[B79] Prock K. A. Kennedy A. C. (2020). Characteristics, experiences, and service utilization patterns of homeless youth in a transitional living program: Differences by LGBQ identity. *Children Youth Serv. Rev.* 116:105176. 10.1016/j.childyouth.2020.105176

[B80] Proulx C. N. Coulter R. W. Egan J. E. Matthews D. D. Mair C. (2019). Associations of lesbian, gay, bisexual, transgender, and questioning–inclusive sex education with mental health outcomes and school-based victimization in US high school students. *J. Adolesc. Health* 64 608–614. 10.1016/j.jadohealth.2018.11.012 30691941 PMC6478545

[B81] Pullen Sansfaçon A. Kirichenko V. Holmes C. Feder S. Lawson M. L. Ghosh S. (2020). Parents’ journeys to acceptance and support of gender-diverse and trans children and youth. *J. Fam. Iss.* 41 1214–1236.

[B82] Ramasamy V. R. (2020). “Young, disabled and LGBT+ identities,” in *Young, Disabled and LGBT+: Voices, Identities and Intersections*, eds Toft A. Franklin A. (Abingdon: Taylor & Francis), 159–178.

[B83] Reyes M. E. S. Davis R. D. Yapcengco F. L. Bordeos C. M. M. Gesmundo S. C. Torres J. K. M. (2020). Perceived Parental Acceptance, Transgender Congruence, and Psychological Well-Being of Filipino Transgender Individuals. *North Am. J. Psychol.* 22 135–152.

[B84] Savin-Williams R. C. (2020). “Coming out to parents and self-esteem among gay and lesbian youths,” in *Homosexuality and the Family*, ed. Bozett F. W. (London: Routledge), 1–35. 10.1300/J082v18n01_01 2794494

[B85] Simons J. D. Russell S. T. (2021). Educator interaction with sexual minority youth. *J. Gay Lesb. Soc. Serv.* 2021 1–24. 10.1080/10538720.2021.1897052 34733062 PMC8562320

[B86] Skovdal M. Campbell C. (2015). Beyond education: What role can schools play in the support and protection of children in extreme settings? *Int. J. Educat. Dev.* 41 175–183. 10.1016/j.ijedudev.2015.02.005

[B87] Standley C. J. (2020). Expanding our paradigms: Intersectional and socioecological approaches to suicide prevention. *Death Stud.* 2020 1–9. 10.1080/07481187.2020.1725934 32048555

[B88] Stones S. Glazzard J. (2020). Tales From the Chalkface: Using Narratives to Explore Agency, Resilience, and Identity of Gay Teachers. *Front. Sociol.* 5:52. 10.3389/fsoc.2020.00052 33869459 PMC8022664

[B89] Strauss P. Cook A. Winter S. Watson V. Wright Toussaint D. Lin A. (2020). Mental health issues and complex experiences of abuse among trans and gender diverse young people: findings from trans pathways. *LGBT Health* 7 128–136. 10.1089/lgbt.2019.0232 32155380

[B90] Tavarez J. (2020). “I can’t quite be myself”: Bisexual-specific minority stress within LGBTQ campus spaces. *J. Divers. Higher Educat.* [Preprint].

[B91] Tollemache N. Shrewsbury D. Llewellyn C. (2021). Que (e) rying undergraduate medical curricula: a cross-sectional online survey of lesbian, gay, bisexual, transgender, and queer content inclusion in UK undergraduate medical education. *BMC Med. Educat.* 21 1–12. 10.1186/s12909-021-02532-y 33579262 PMC7881554

[B92] Toomey R. B. Ryan C. Diaz R. M. Russell S. T. (2011). High school gay–straight alliances (GSAs) and young adult well-being: An examination of GSA presence, participation, and perceived effectiveness. *Appl. Dev. Sci.* 15 175–185. 10.1080/10888691.2011.607378 22102782 PMC3217265

[B93] Town R. Hayes D. Fonagy P. Stapley E. (2021). A qualitative investigation of LGBTQ+ young people’s experiences and perceptions of self-managing their mental health. *Eur. Child Adolesc. Psychiatry* 2021 1–14. 10.1007/s00787-021-01783-w 33903961 PMC8075021

[B94] Travers Á Armour C. Hansen M. Cunningham T. Lagdon S. Hyland P. (2020). Lesbian, gay or bisexual identity as a risk factor for trauma and mental health problems in Northern Irish students and the protective role of social support. *Eur. J. Psychotraumatol.* 11:1708144. 10.1080/20008198.2019.1708144 32128041 PMC7034482

[B95] Van Bergen D. D. Bos H. M. W. Van Lisdonk J. Keuzenkamp S. Sandfort T. G. M. (2013). Victimization and suicidality among Dutch lesbian, gay, and bisexual youths. *Am. J. Public Health* 103 70–72. 10.2105/AJPH.2012.300797 23153134 PMC3518328

[B96] Walls N. E. Atteberry-Ash B. Kattari S. K. Peitzmeier S. Kattari L. Langenderfer- Magruder L. (2019). Gender Identity, Sexual Orientation, Mental Health, and Bullying as Predictors of Partner Violence in a Representative Sample of Youth. *J. Adolesc. Health* 64 86–92. 10.1016/j.jadohealth.2018.08.011 30392863

[B97] Willging C. E. Green A. E. Ramos M. M. (2016). Implementing school nursing strategies to reduce LGBTQ adolescent suicide: a randomized cluster trial study protocol. *Implement. Sci.* 11 1–11. 10.1186/s13012-016-0507-2 27770819 PMC5075193

[B98] Wilson B. D. M. Cooper K. Kastanis A. Nezhad S. (2014). *Sexual and Gender Minority Youth in Foster Care: Assessing Disproportionality and Disparities in Los Angeles.* Los Angeles, CA: The William Institute.

[B99] Wilson C. Cariola L. A. (2020). LGBTQI+ youth and mental health: a systematic review of qualitative research. *Adolesc. Res. Rev.* 5 187–211. 10.1111/appy.12199 26238088

[B100] Woodford M. R. Kulick A. Garvey J. C. Sinco B. R. Hong J. S. (2018). LGBTQ policies and resources on campus and the experiences and psychological well-being of sexual minority college students: Advancing research on structural inclusion. *Psychol. Sex. Orientat. Gender Divers.* 5:445. 10.1037/sgd0000289

[B101] Ybarra M. L. Mitchell K. J. Kosciw J. G. Korchmaros J. D. (2015). Understanding linkages between bullying and suicidal ideation in a national sample of LGB and heterosexual youth in the united states. *Prevent. Sci.* 16 451–462. 10.1007/s11121-014-0510-2 25322949

